# Influence of depressive disorders, stress, and personality traits on quality of life after cochlear implantation

**DOI:** 10.1007/s00405-023-08284-3

**Published:** 2023-11-02

**Authors:** Susen Lailach, Paula Stephan, Johanna Martin, Thomas Zahnert, Marcus Neudert

**Affiliations:** Faculty of Medicine Carl Gustav Carus, Department of Otorhinolaryngology, Head and Neck Surgery, Technische Universität Dresden, Fetscherstraße 74, 01307 Dresden, Saxony Germany

**Keywords:** Mental health, Patient-reported outcome measures, Disease-specific health-related quality of life, Depressiveness, Hearing disorders, Rehabilitation

## Abstract

**Purpose:**

This study aimed to determine whether preoperative depressiveness, stress, and personality influence quality of life (QOL) after cochlear implant (CI) surgery.

**Methods:**

In this prospective study, 79 patients undergoing CI surgery were evaluated preoperatively and 12 months postoperatively. Disease-specific QOL was assessed with the Nijmegen Cochlear Implant Questionnaire (*NCIQ*) and general QOL with the WHOQOL-BREF. Depressiveness and stress were assessed with the Patient Health Questionnaire (PHQ-D). The Charlson Comorbidity Index (CCI) was used to classify comorbidities. The Big Five Personality Test (B5T) was used to assess the basic personality dimensions. Speech comprehension was evaluated in quiet with the Freiburg monosyllable test and in noise with the Oldenburg sentence test.

**Results:**

After CI surgery, the total NCIQ score improved significantly (Δ 17.1 ± 14.7, *p* < 0.001). General QOL (WHOQOL-BREF, Δ 0.4 ± 9.9, *p* = 0.357), stress (Δ 0.25 ± 3.21, *p* = 0.486), and depressiveness (Δ 0.52 ± 3.21, *p* = 0.121) were unaffected by CI surgery. Patients without elevated depressiveness (*p* < 0.01) or stress (*p* < 0.001) had significantly better total NCIQ scores. The results of the multiple regression analyses show that, after adjusting for the CCI, personality, age, and mental health stress (ß = − 0.495, *p* < 0.001) was significantly associated with postoperative NCIQ outcome scores. Depressiveness and neuroticism had the strongest influence on the generic QOL (ß = − 0.286 and ß = − 0.277, *p* < 0.05).

**Conclusion:**

Stress symptoms and personality traits are significant predictive factors for disease-specific QOL, as well as hearing status. This should be considered in the preoperative consultation and in optimizing the rehabilitation process.

## Introduction

Worldwide, 60 million people suffer from severe or profound hearing loss [1]. Today, it is estimated that inadequately treated hearing loss results in a global financial burden of more than 980 billion US dollars [1]. However, as well as the financial burden, a mainly individual psychosocial workload (stress, mental workload, emotional workload) exists due to the resulting communication impairment and limited social interaction, which is difficult to quantify [1]. Patient-reported outcome measures (PROMs) and, particularly, disease-specific quality of life (QOL) measures have been increasingly developed in recent years to address this problem. These instruments are used to not only measure the psychosocial strain of hearing loss but also evaluate the effectiveness of treatment procedures for hearing loss. Today, cochlear implants (CIs) are indispensable in the auditory rehabilitation of severely hard-of-hearing patients. Due to the rapid development of implant technology, hearing outcomes have improved significantly [2]. Nevertheless, clinical experience in daily life shows that patients do not always reflect equally high gains in QOL despite very good speech understanding with the CI.

While QOL is often neglected as an outcome parameter in otorhinolaryngology, it is a fixed quality indicator in the CI treatment process and is firmly implemented in the current guidelines and national CI registry [3, 4]. Numerous patient studies have demonstrated improvement in QOL after cochlear implantation. Noticeably, this improvement does not always correlate significantly with improved hearing outcomes [5, 6]. For example, while Cieśla et al. (2016), Hirschfelder et al. (2008), and Walia et al. (2023) identified a significant association of speech comprehension and QOL, this could not be confirmed in research by Capretta and Moberly (2016), an Vasil et al. (2020) [5–9]. The evaluation of the potential influence of age also shows inhomogeneous results. While Hallberg et al. (2005) demonstrated negative age effects, le Roux et al. (2017) found similar QOL scores for older and younger adults, Olze et al. (2012) even showed a better QOL with increasing age [10–12].

These facts lead to the hypothesis that patient-associated factors may also determine QOL in CI patients. Therefore, further exploration of potential influencing factors, such as depressiveness, comorbidities, personality traits, and hearing outcome, is needed to improve the QOL of patients with CI. Knowledge of potential patient-related factors influencing QOL in patients with CI can optimize not only the preoperative counseling of patients but also the setting of rehabilitation goals and the individualization of rehabilitation content.

For rehabilitative middle ear surgery, it was recently shown that depressiveness is the primary factor influencing postoperative QOL [13]. Other psychological disorders have been identified as influencing treatment outcomes. These include, for example, increased stress levels, anxiety disorders, and personality traits [14–16]. Studies by Olze et al. (2011), Knopke et al. (2019), and Brüggemann et al. (2017) have also shown a negative effect of depressive disorders on QOL in CI patients [17–19]. Likewise, these mentioned studies showed also a negative influence of increased stress and anxiety on postoperative QOL. For increased anxiety in particular, there is a strong association with depressive disorders in CI patients [19]. In addition, previous studies show a strong negative influence of increased tinnitus burden on QOL after CI fitting, with a strong multicollinearity of psychological comorbidities and tinnitus [19, 20].

Some studies have shown that personality traits may play an important role for different diseases in assessing symptoms, treatment decisions, and treatment outcomes [21–23]. Personality traits are lifelong traits reflecting someone’s attitude toward a particular situation. In patients with CIs, personality traits can play a role in the treatment process in many ways. For example, they may influence the patient’s willingness to engage in CI surgery and subsequent rehabilitation. Additionally, personality traits are associated with neuropsychiatric disorders such as anxiety and depression. These psychological aspects may impact coping strategies during the treatment process. While the influence of personality traits, in particular the negative influence of neuroticism, on the perceived QOL has already been proven in studies in other medical fields [14], only sporadic studies exist for CI patients so far [24]. For example, Muigg et al. (2019) were also able to prove the negative effect of neuroticism on QOL after CI treatment. However, in this study, the hypothesized relationship between personality traits and depressiveness was not addressed.

Finally, in the mentioned studies the relationship between psychological and audiological factors and QOL in CI patients was preferably considered unidimensionally. Especially for psychological comorbidities and personality traits, correlation structures can be assumed, which have also been proven in psychological studies [25, 26]. For CI patients, however, an elaboration of the correlations is still pending. However, an exclusive consideration of psychological aspects does not reflect the complex construct of QOL. Especially somatic complaints have to be considered in this context. To what extent concomitant diseases influence QOL in CI patients has not been investigated in studies so far. However, studies from other medical fields imply a negative influence of increased comorbidities on QOL in other treatment processes [27, 28]. In addition to somatic and psychological aspects, social factors are also important in CI care.

In a multivariate analysis of the influence of social and demographic factors on QOL after CI care, Hallberg et al. (2005) identified age, support of others, restricted participation and attitudes as significant influencing factors [10]. In this study, however, somatic and psychological disorders were not taken into account. The aim of our study was to work out the correlations between these two constructs within the framework of a multivariate analysis. Therefore, the present study aimed to analyze the influence of personality traits, depressive symptoms, stress, comorbidity and hearing outcome on QOL after cochlear implantation. The empirical selection of the factors to be considered was based on the literature mentioned above, taking into account already known associations of individual factors. After elaboration of the influence of psychological and physical influencing factors, the integration of already identified social factors should be aimed at in further studies.

## Patients and methods

### Study design and population

**Fig. 1:**
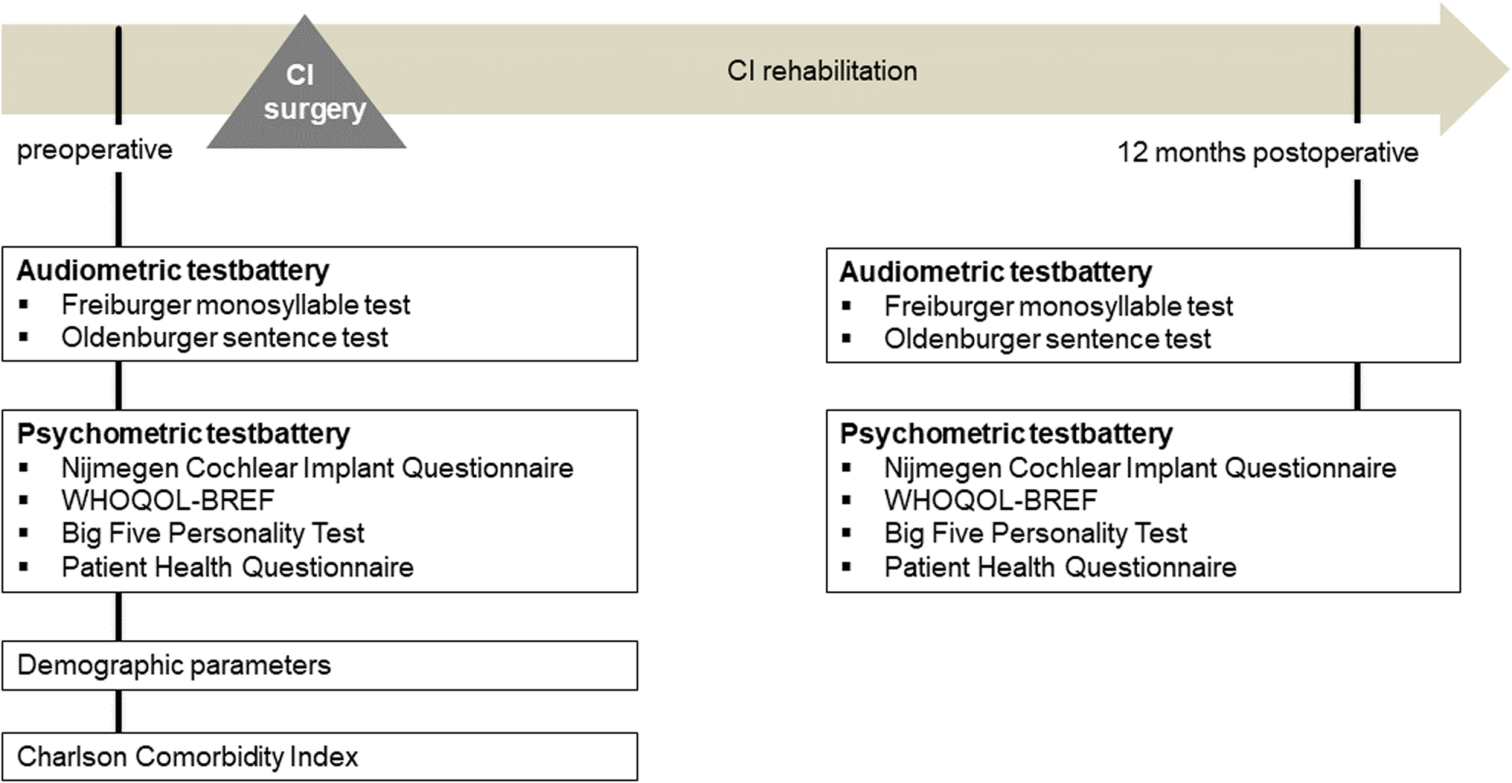
Study protocol: measurement time points and measurement instruments

Adults with postlingual deafness and planned cochlear implantation were enrolled prospectively between July 2020 and July 2021. Preoperative audiological was performed during the complex outpatient diagnostics before cochlear implantation. Psychometric measurements were performed on the day of preoperative surgery preparation 7–14 days before surgery. A treatment evaluation was performed 12 months postoperatively (Fig. 1). Patients who missed the control visit after 12 months were excluded.

All patients who were scheduled for cochlear implantation during the observation period were included. Inclusion criteria were postlingual deafness, German native language proficiency, full legal capacity, and age greater than 18 years. Patients with prelingual deafness were excluded. Furthermore, patients who did not attend the 12-month appointment in the rehabilitation program were excluded. Assessment of language status was routinely performed during evaluation by a speech therapist in an exploratory interview.

CI rehabilitation was performed as inpatient procedure in our clinic despite COVID-19 pandemic. Telemedical or outpatient procedures were not used.

Institutional review board approval was obtained before study initiation by the local ethical review committee (EK-247062020). All patients gave their informed consent. The study was conducted in accordance with the Declaration of Helsinki 1964.

### Audiological assessment

The Freiburg monosyllabic word test was used to determine the preoperative speech recognition scores in quiet at 65 dB SPL and 80 dB SPL with an optimized hearing aid preoperatively and 12 months after cochlear implantation. Depending on the audiological results, the hearing aids were readjusted by the hearing care professional. The adjustment was then checked in the clinic by means of in situ measurement or hearing aid test box.

The contralateral ear was masked in patients with residual hearing. Up to now, there is no national or international standardization of masking for speech audiometry in free field condition. In our department, patients with residual hearing on the contralateral side better than 60 dB in the pure tone average at 0.5, 1. 2 and 4 kHz are individually masked. For this purpose, the lowest necessary masking level is determined individually for each patient without using the speech processor, and the maximum masking level can be 90 dB.

Speech recognition in background noise was tested with the Oldenburg sentence test (OLSA). The 50% speech reception threshold (SRT) was determined adaptively with a fixed noise level of 65 dB SPL. Under the presentation condition S0N0, speech and noise were displayed from the front at an angle of 0°. The OLSA was performed preoperatively and postoperatively in the bilateral aided configuration.

### Quality of life measurement

Disease-specific QOL measurement was performed using the validated and disease-specific Nijmegen Cochlear Implant Questionnaire (NCIQ) in German. The World Health Organization Quality of Life: Best Available Techniques Reference (WHOQOL-BREF) was used to determine general QOL.

#### Nijmegen cochlear implant questionnaire (NCIQ)

The NCIQ was developed and validated to measure disease-specific QOL in adults with CIs [29]. The German version of the NCIQ was validated by Plath et al. (2022) [30]. The questionnaire is based on three superordinate domains and six subdomains, which analyze the QOL according to aspects of physical (subdomains: basic sound perception, advanced sound perception, speech production), mental (subdomain: self-esteem), and social functionality (subdomains: activity limitations, social interactions). Five defined answer categories are available for each item, as well as a sixth category for the case of a question being impossible to answer. For the evaluation, it is necessary to consider 27 inversely scored items. The 10 items in each subdomain are then summed and divided by the number of completed items. A higher score represents a better evaluation of disease-specific QOL.

#### The world health organization quality of life—best available techniques reference (WHOQOL-BREF)

WHOQOL-BREF is the abbreviated version of WHOQOL-100 [31]. With only 26 rather than 100 items, the WHOQOL-BREF is also suitable for patients with limited resilience and time. The German version from 2002 was used in this study. The questionnaire items with 5-point responses are grouped into four domains: physical health, psychological health, social relationships, and environment. The mean values of the answers are calculated and then multiplied by four to determine the score of a domain. This results in values between 4 and 20 for each domain, which are normalized to a scale from 0 to 100. The total score is calculated from the mean value of all subscores. Higher scores indicate better generic QOL.

### Personality assessment

The Big Five Personality Test (B5T) was used to determine the expression of the five basic personality dimensions (neuroticism, extraversion, conscientiousness, agreeableness, and openness; Table 1). The German B5T is a reliable and validated questionnaire that can be used for clinical diagnostics [32]. The test contains 10 items per personality structure, which are rated by the test participants from 1 (does not apply at all) to 4 (applies exactly). The scores of the 10 items of a scale are added to give raw scores between 10 and 40, which are then transferred to stanine norm scores using a suitable norm score table. The norm value tables consider the gender (division into male and female) and age (under 20 years, 20 to 50 years, and over 50 years) of the test subjects. The level of the stanine norms characterizes the expression of the respective personality dimension, whereby stanine norm values can range from 1 (extremely low expression) to 9 (extremely strong expression).

**Table 1:** Interpretation of high stanine norm scores (values from 7 to 9) of the Big 5 Personality Test (B5T) for the individual personality dimensions

Personality dimension	Characteristics of persons with above-average expression of the dimension (stanine norm values 7–9)
Neuroticism	Anxiousness, nervousness, insecurity, self-doubt
Extraversion	Sociability, talkativeness, sociability, activity
Conscientiousness	Neatness, organization, structure
Agreeableness	Politeness, courtesy, popularity, sympathy
Openness	Curiosity, thirst for knowledge, creativity, interest

### Psychological health

The German version of the Patient Health Questionnaire (PHQ-D) was used to assess psychological health [33]. The PHQ-D has been developed to screen for mental disorders in primary care. It is based on diagnostic criteria from the fourth edition of the American Psychiatric Association’s Diagnostic and Statistical Manual of Mental Disorders (DSM-IV) [34].

#### Depressiveness

The depression module (PHQ-9) of the PHQ-D is a screening tool used in numerous patient populations to quantify the severity of patient-reported depressive symptoms (“depressiveness”). Patients rate the occurrence of depressive symptoms within the last 2 weeks on a 4-point scale from 0 (“not at all”) to 3 (“nearly every day”). The severity of depressive symptoms was analyzed as a continuous score from 0 (none) to 27 (severe). A scale value below 5 practically corresponds to the absence of a depressive disorder.

#### Stress

The PHQ stress module measures psychosocial stress during the last month using 10 items, including health, work/financial, social, and traumatic stress. Patients rate the severity of stress on a scale of 0 (“not at all bothered”) to 2 (“bothered a lot”). The severity of stress was analyzed on a continuous scale from 0 (none) to 20 (severe). A scale value below 5 indicates only minimally present psychosocial stress factors.

### Comorbidity

The Charlson Comorbidity Index (CCI, Table 2) comprises 17 comorbidities, which are weighted (from 1 to 6) based on the adjusted risk of mortality or resource use [35]. The sum of all the weights results in a single comorbidity score for each patient.

**Table 2:** Charlson comorbidity index (CCI)

Comorbid conditions	CCI weights
Myocardial infarction	0
Congestive heart failure	2
Peripheral vascular disease	0
Cerebrovascular disease	0
Dementia	2
Chronic pulmonary disease	1
Rheumatic disease	1
Peptic ulcer disease	0
Mild liver disease	2
Diabetes without chronic complications	0
Diabetes without chronic complications	1
Paraplegia or hemiplegia	2
Renal disease	1
Any malignancy without metastasis	2
Moderate or severe liver disease	4
Metastastic malignoma	6
AIDS /HIV	4

### Statistical analysis

Descriptive statistics, including the mean and standard deviation (SD), were used to represent demographic and outcome data. The normality of the outcome scores was assessed with the Kolmogorov–Smirnov test. For normally distributed outcome scores, a paired t-test was used to compare the mean scores pre- and postoperatively. QOL score were compared using a one-way analysis of variance (ANOVA) for the three groups and Student’s *t* test for two groups. Multiple linear regression analyses were conducted to determine the effect of preoperative psychological health and personality on outcome scores, controlling for comorbidity (CCI), age, and hearing status. The factors included were selected empirically on the basis of the literature research presented in the introduction, taking into account previously described correlations. A *p*-value < 0.05 was considered statistically significant. Statistical analyses were performed using IBM SPSS Statistics 28 (SPSS Inc., Chicago, IL, USA).

## Results

**Fig. 2:**
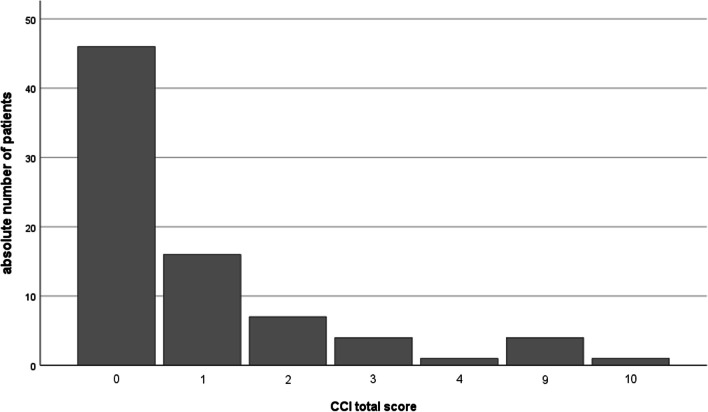
Description of the comorbidity of the included patient group based on the Charlson Comorbidity Index (CCI): absolute number of patients (*n* = 79). Higher values are associated with greater comorbidity

This study included 79 patients (35 males and 44 females) with a mean age of 57.56 ± 15.96 years (range 21–86 years). The dropout rate was 21.8% (22/101 patients). Eleven patients (10.89%) did not want to retake the psychometric measurements at the follow-up appointment for personal reasons. Nine patients (8.91%) did not show up for the 12-month appointment due to the COVID-19 pandemic, but attended later appointments. In 2 patients (1.98%), the implant had to be changed due to an implant defect and CI rehabilitation had to be restarted. There were no non-users among the patients analyzed. The status of the contralateral ear is shown in Table 3. The mean CCI score was 1.16 ± 2.30 (Fig. 2).

**Table 3:** Demographic data (*n* = 79)

Parameter		Frequency out of *n* = 79 (absolute number)
Gender	Female	55.7% (44)
	Male	44.3% (35)
Side	Left	59.5% (47)
	Right	40,5% (32)
Hearing Status	SSD	21.5% (17)
	AHL	21.5% (17)
	Bilateral hearing loss	57.0% (45)
Hearing solution contralateral ear	Hearing Aid	39.7% (31)
	Cochlear implant	33.3% (26)
	Active Middle Ear Implant or Bone Conduction Implant	14.1% (11)
	None	12.8% (10)
Implant company	Cochlear	59.5% (47)
	MED-EL	34.2% (27)
	Advanced Bionics	6.3% (5)

### Audiological results

The speech perception in quiet, measured at 65 dB SPL, improved from 7.1 ± 16.0% to 57.9 ± 27.7% (*p* < 0.001) after cochlear implantation. The word recognition score measured at 80 dB SPL improved from 16.3 ± 26.3% to 70.1 ± 23.5% (*p* < 0.001) after surgery. The SRT decreased from 3.56 ± 7.42 to 0.50 ± 4.03 dB (*p* < 0.001).

### Quality of life

**Fig. 3:**
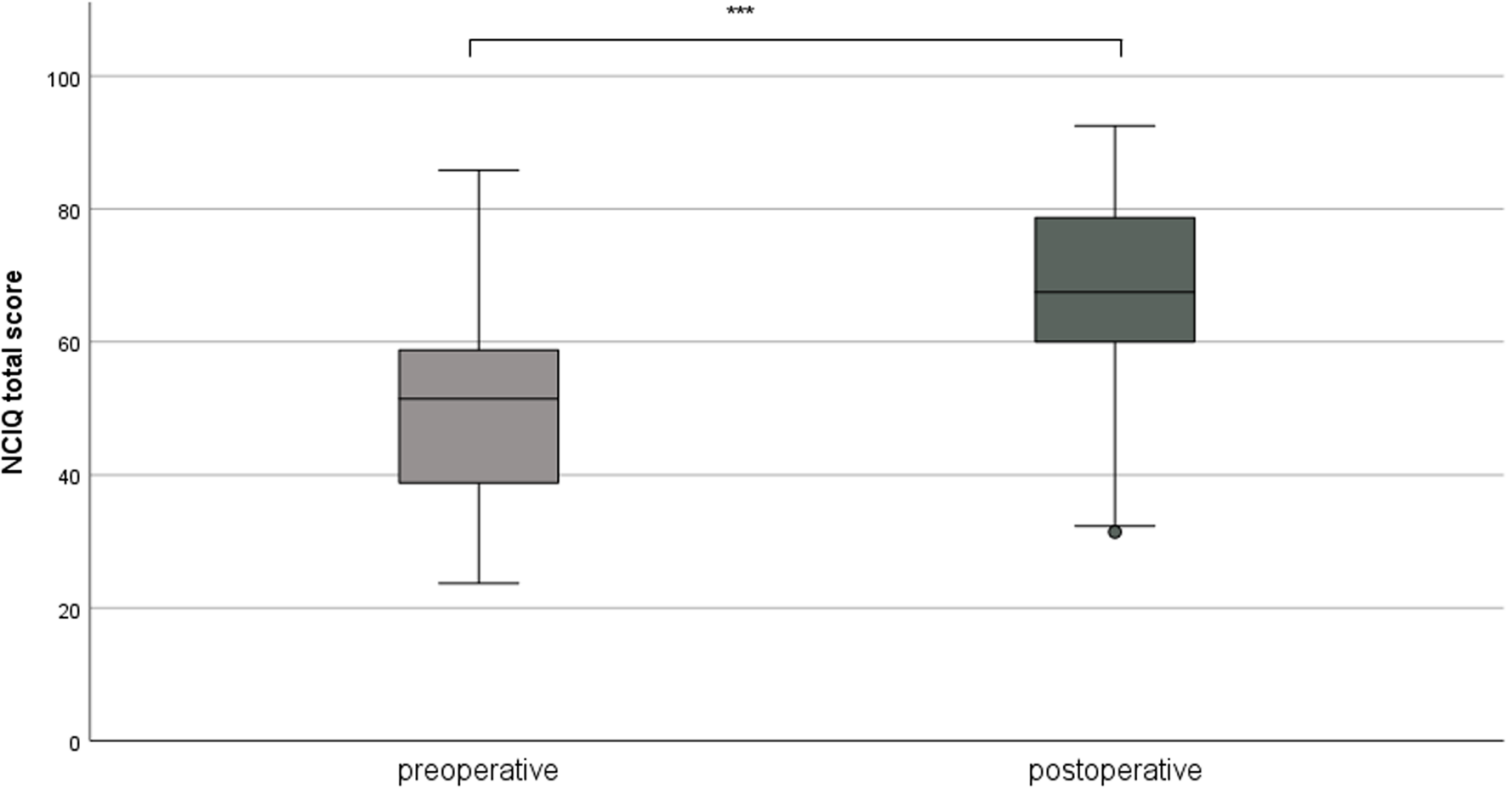
NCIQ total score: comparison of pre- and postoperative results (*n* = 79). Higher values indicate a better QOL; ***p ≤ 0.001 for preoperative versus postoperative

**Fig. 4:**
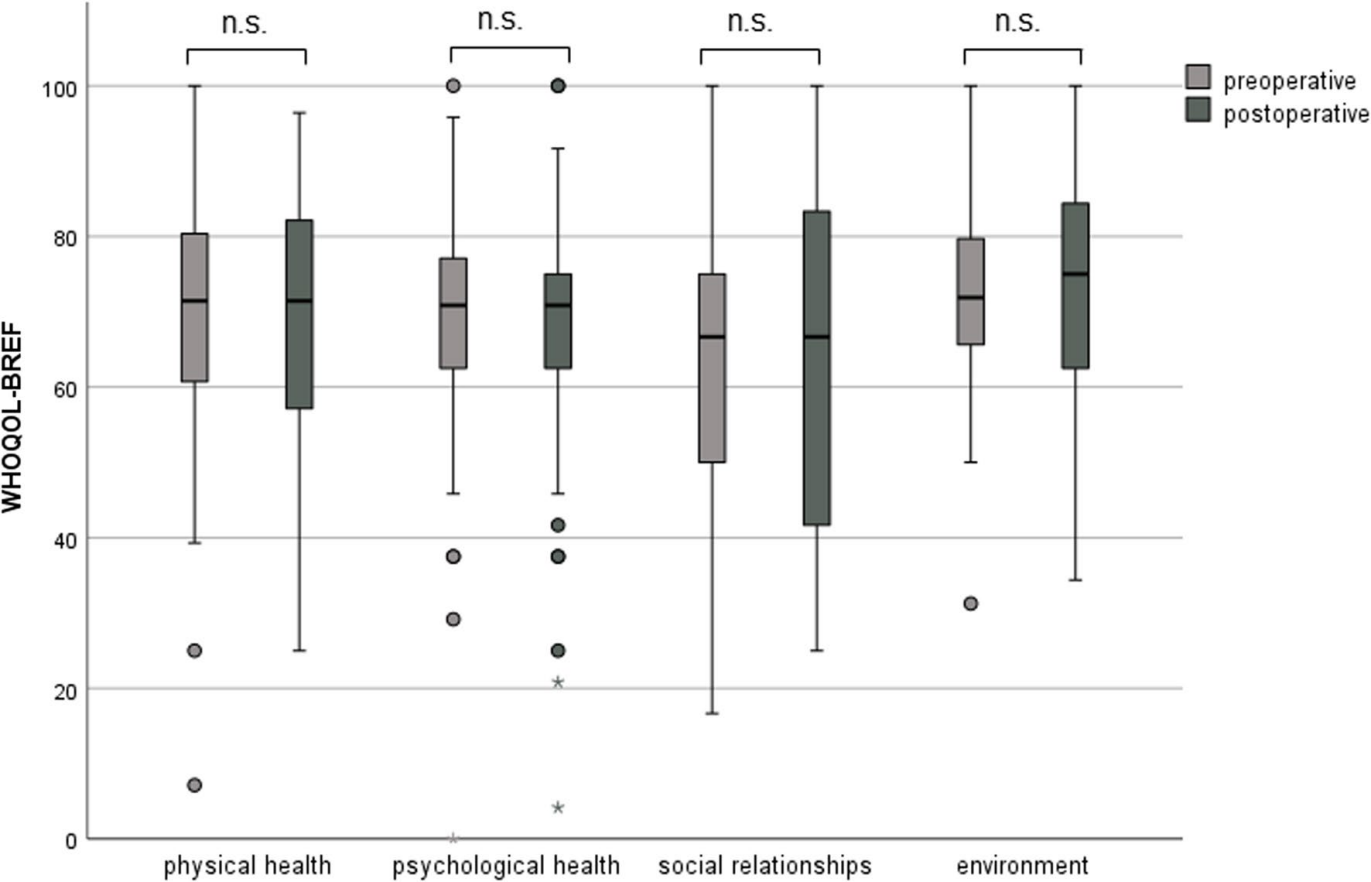
WHOQOL-BREF: comparison of pre- and postoperative results (*n* = 79); Higher values indicate a better QOL; n.s.: not significant (*p* > 0.05)

Cochlear implantation resulted in a significantly better total NCIQ score (50.1 ± 14.1 versus 67.6 ± 13.7, *p* < 0.001) postoperatively (Fig. 3). However, when comparing the preoperative and postoperative results, no significant change was observed in the WHOQOL-BREF total score (68.9 ± 12.6 versus 68.5 ± 14.5, *p* = 0.357) or in the individual subscores (Fig. 4). There were no gender-related differences in pre- and postoperative NCIQ and WHOQOL-BREF scores (Table 4). Significant improvement in NCIQ total scores, but not in WHOQOL-BREF total scores, was found for the senior group and the non-senior patient group. Postoperative disease-specific QOL (NCIQ) was significantly better in the non-elderly patient group than in the senior group (Table 4). No significant difference between the two groups could be determined for generic QOL (Table 4). When considering patients with different types of hearing loss (SSD, AHL and bilateral hearing loss), all three groups of patients showed significant improvement in NCIQ scores. However, only SSD patients showed a significant improvement in generic QOL. While preoperatively there was a significant difference in disease-specific QOL between the SSD and bilateral hearing loss groups, postoperatively there were no significant differences in either NCIQ or WHOQOL between the three groups analyzed (Table 4).

**Table 4:** Comparison of pre- and postoperative disease-specific QOL and generic QOL stratified by gender, type of hearing loss, and psychological comorbidity

	NCIQ		WHOQOL-BREF	
	Preoperative	Postoperative	Preoperative	postoperative
Gender				
Male (*n* = 35)	49.8 ± 13.1	64.2 ± 12.9 ***	68.6 ± 10.5	66.9 ± 13.46
Female (*n* = 44)	50.3 ± 15.1	69.7 ± 13.7 ***	69.0 ± 14.2	69.6 ± 15.3
Age				
< 65 years (n = 49)	51.1 ± 14.8	70.0 ± 13.3***	68.8 ± 14.5	68.3 ± 16.8
≥ 65 years (*n* = 30)	48.5 ± 12.9	62.7 ± 12.7*** ^##a^	68.8 ± 8.7	68.6 ± 9.9
Kind of hearing loss				
SSD (*n* = 17)	59.9 ± 11.2^##b^	74.4 ± 12.7***	71.7 ± 7.5	75.0 ± 16.0 *
AHL (*n* = 17)	51.7 ± 12.1	65.0 ± 12.6 ***	66.8 ± 10.5	64.0 ± 13.2
Bilateral hearing loss (*n* = 45)	46.2 ± 14.2^##b^	65.6 ± 13.6 ***	68.5 ± 11.2	67.6 ± 13.9
Depressiveness				
Non-elevated symptoms^1^ (*n* = 42)	53.9 14.1	71.7 11.6 ***	74.7 ± 10.2	74.0 ± 13.5
Elevated symptoms^2^ (*n* = 37)	45.8 13.0^#c^	62.0 13.8 *** ^###c^	62.2 ± 11.8 ^###c^	62.1 ± 13.1 ^###c^
Stress Stress				
Non-elevated stress level ^3^ (*n* = 54)	53.3 ± 14.0	70.8 ± 11.7 ***	72.3 ± 10.8	72.2 ± 12.8
Elevated stress level ^4^ (*n* = 25)	43.1 ± 11.8 ^##d^	59.2 ± 13.9 *** ^###d^	61.3 ± 13.0 ^###d^	60.1 ± 14.7 ^###d^

*AHL* asymmetric hearing loss (defined as an interaural asymmetry of more than 30 dB, whereas the better hearing ear has less or equal to 60 dB HL and more than 30 dB HL at one or more frequencies across the pure tone average)

*SSD* single sided deafness (unilateral deafness and contralateral normal hearing with a pure tone average less or equal to 30 dB HL)

^1^PHQ 9 < 5, ^2^PHQ 9 ≥ 5, ^3^PHQ stress module < 5, ^4^PHQ stress module ≥ 5

^***^p < 0.001, **p < 0.01, *p < 0.05 (preoperative versus postoperative)

^##a^*p* < 0.001 significant difference between age groups

^## b^*p* < 0.001 significant difference between bilateral hearing loss group and SSD group

^#c^*p* < 0.05, ^###c^*p* < 0.001 significant difference between patient with and without elevated depressive symptoms

^##d^*p* < 0.01, ^###c^*p* < 0.001 significant difference between patient with and without elevated stress level

### Psychological health and personality dimensions

**Fig. 5:**
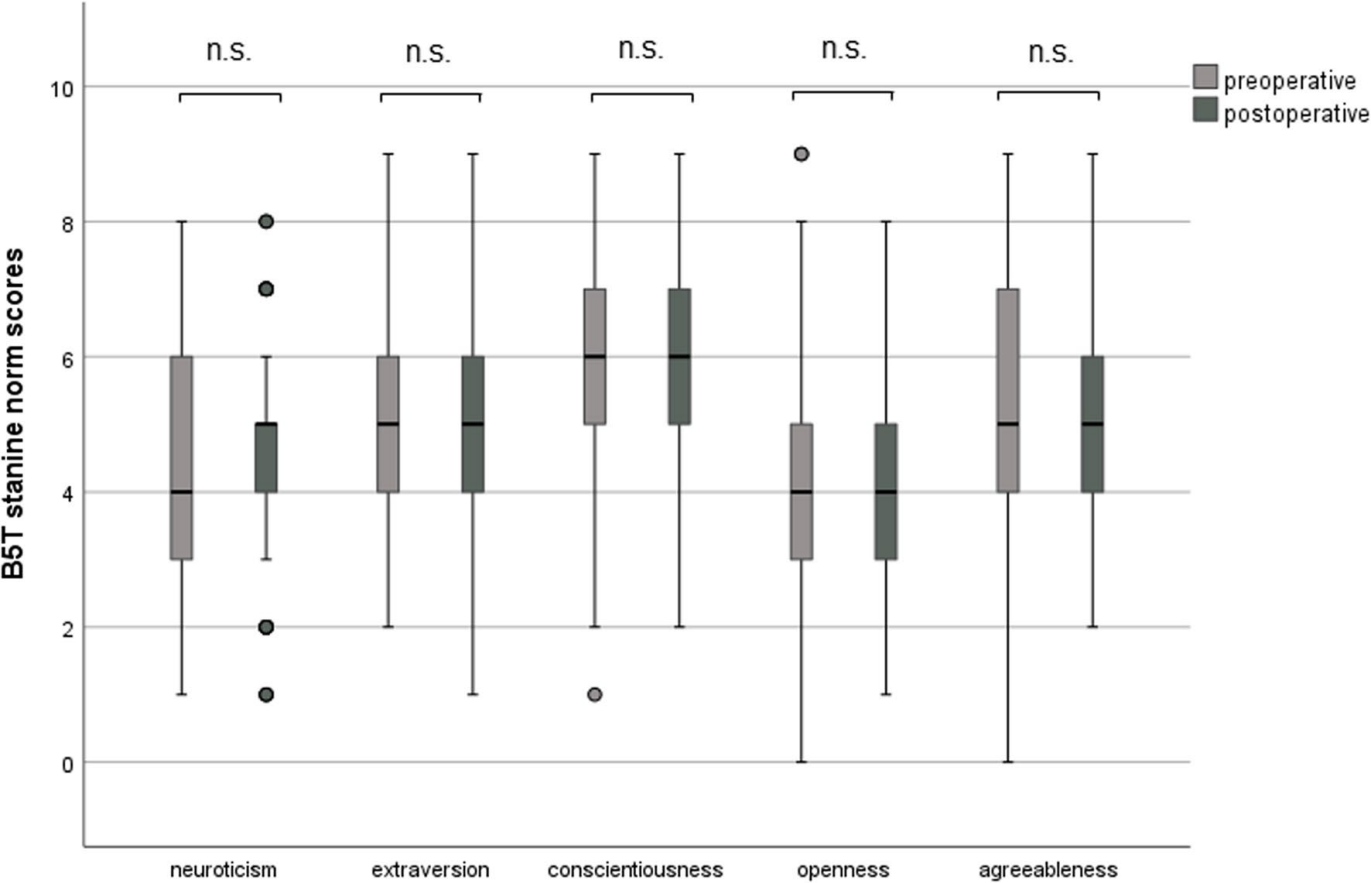
Characteristics of the personality traits of the patient group (*n* = 79) based on the Big Five Personality Test (B5T): presentation of stanine norm scores pre- and postoperatively. Higher scores are associated with a stronger expression of personality traits; n.s.: not significant (*p* > 0.05)

**Fig. 6:**
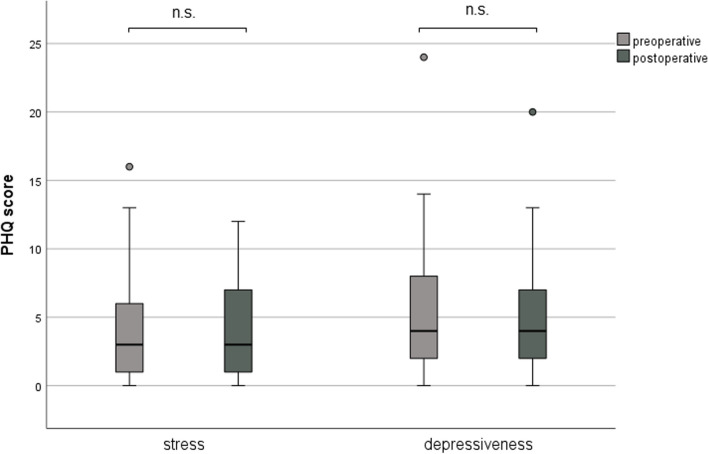
Comparison of depressiveness and stress levels pre- and postoperatively using the PHQ-9 and PHQ stress modules, respectively. Higher scores are associated with higher depressiveness and stress levels; n.s.: not significant (*p* > 0.05)

**Fig. 7:**
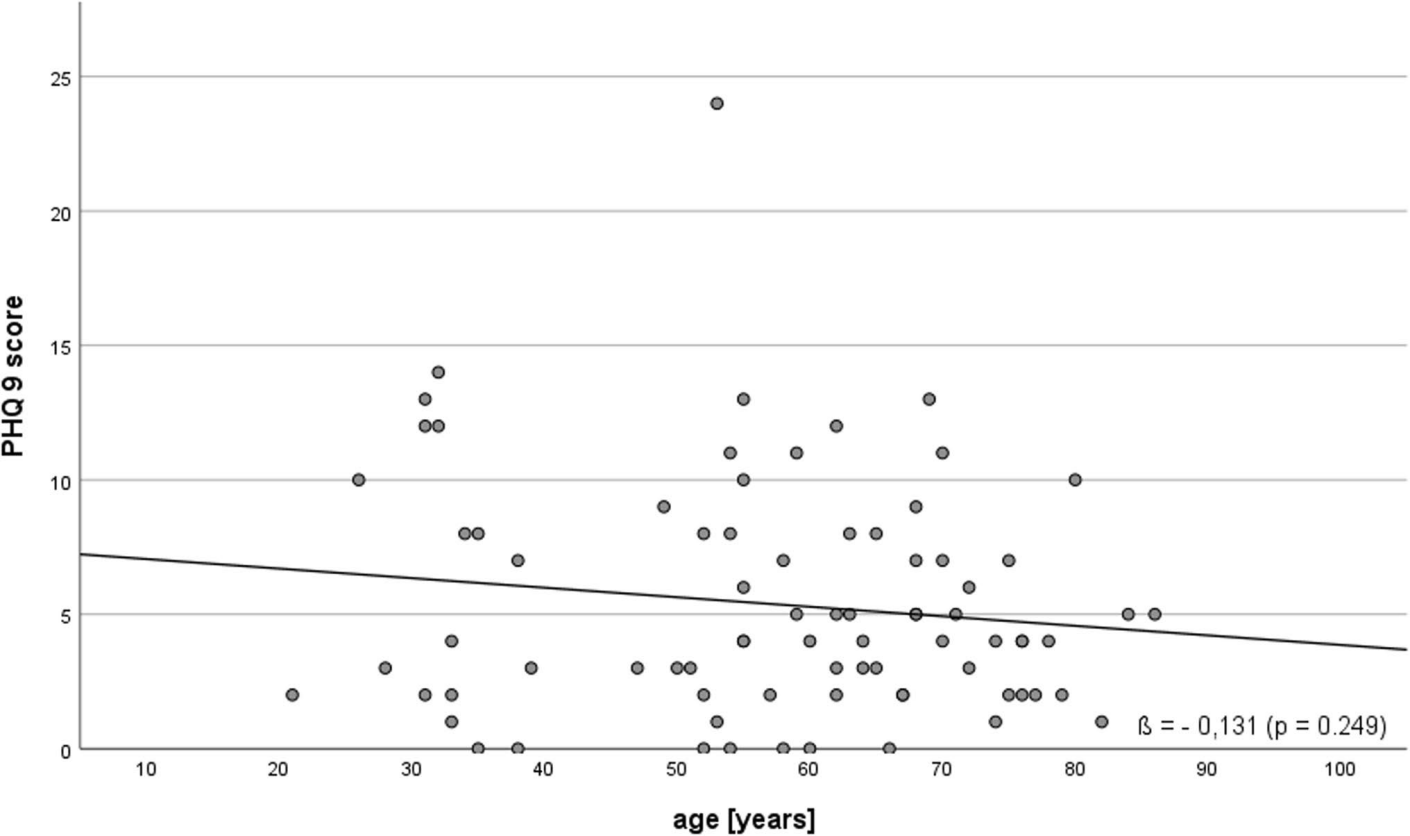
Association of preoperative depressiveness and patient age (total group, *n* = 79)

**Fig. 8:**
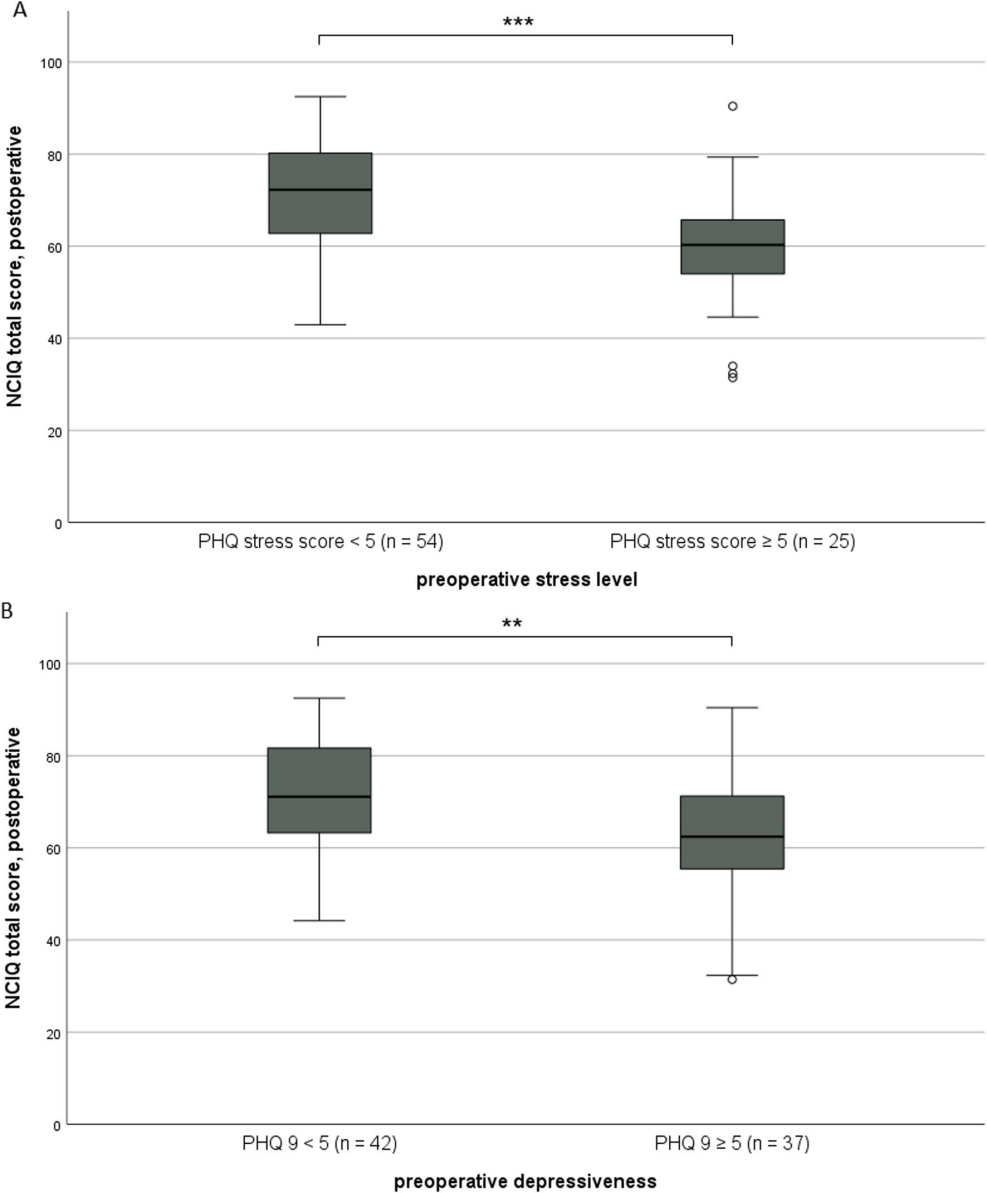
Postoperative assessment of the NCIQ: (**A**) comparison of patients with preoperative minimally pronounced psychosocial stressors (PHQ stress module < 5) versus patients with more pronounced stressors (PHQ stress module ≥ 5); (**B**) comparison of patients with preoperative non-minimal depressive symptoms (PHQ-9 < 5) versus patients with depressive symptoms (PHQ-9 ≥ 5); ***p ≤ 0.001, **p ≤ 0.01

It was shown that the expression of the five analyzed personality traits was not changed significantly by cochlear implantation (Fig. 5). Generally, depressiveness (Δ 0.52 ± 3.21, *p* = 0.121) and stress (Δ 0.25 ± 3.21, *p* = 0.486) measured by PHQ-D scores did not change after cochlear implantation (Fig. 6). In our analyzed group of patients, only a weak, non-significant association of age and depressiveness was found (ß 0.131, *p* = 0.249, Fig. 7). Of the participants, 37 (46.8%) had a PHQ-9 score of 5 or greater, indicating elevated depressive symptoms preoperatively. Twenty-five (31.6%) patients had a stress score exceeding 5, indicating increased stress levels in these patients. Patients with elevated stress symptoms showed significantly lower NCIQ scores than patients without a higher stress load (*p* < 0.01, Fig. 8A). The mean postoperative NCIQ scores were significantly lower in patients with preoperative depressive symptoms than in those without depressive symptoms (*p* < 0.001, Fig. 8B). Disease-specific QOL improved significantly in the patient group with increased depressive symptoms (Δ 16.2 ± 15.5, *p* < 0.001) and in the patient group without increased depressive symptoms (Δ 17.8 ± 14.1, *p* < 0.001). No significant improvement was found for generic QOL in either group (Table 4). Generic QOL was significantly rated worse preoperatively and postoperatively by the patient group with increased depressiveness than by the patients without increased depressive symptoms (Table 4). Patients with increased stress perception (Δ 16.1 ± 15.2, *p* < 0.001) and patients without increased stress level (Δ 17.5 ± 14.5, *p* < 0.001) showed significant improvement in NCIQ total scores but no improvement in generic QOL. The generic QOL was scored significantly worse by the patients with increased stress level than in the patient group without increased stress perception (Table 4).

### Multivariate analysis of influencing factors

**Fig. 9:**
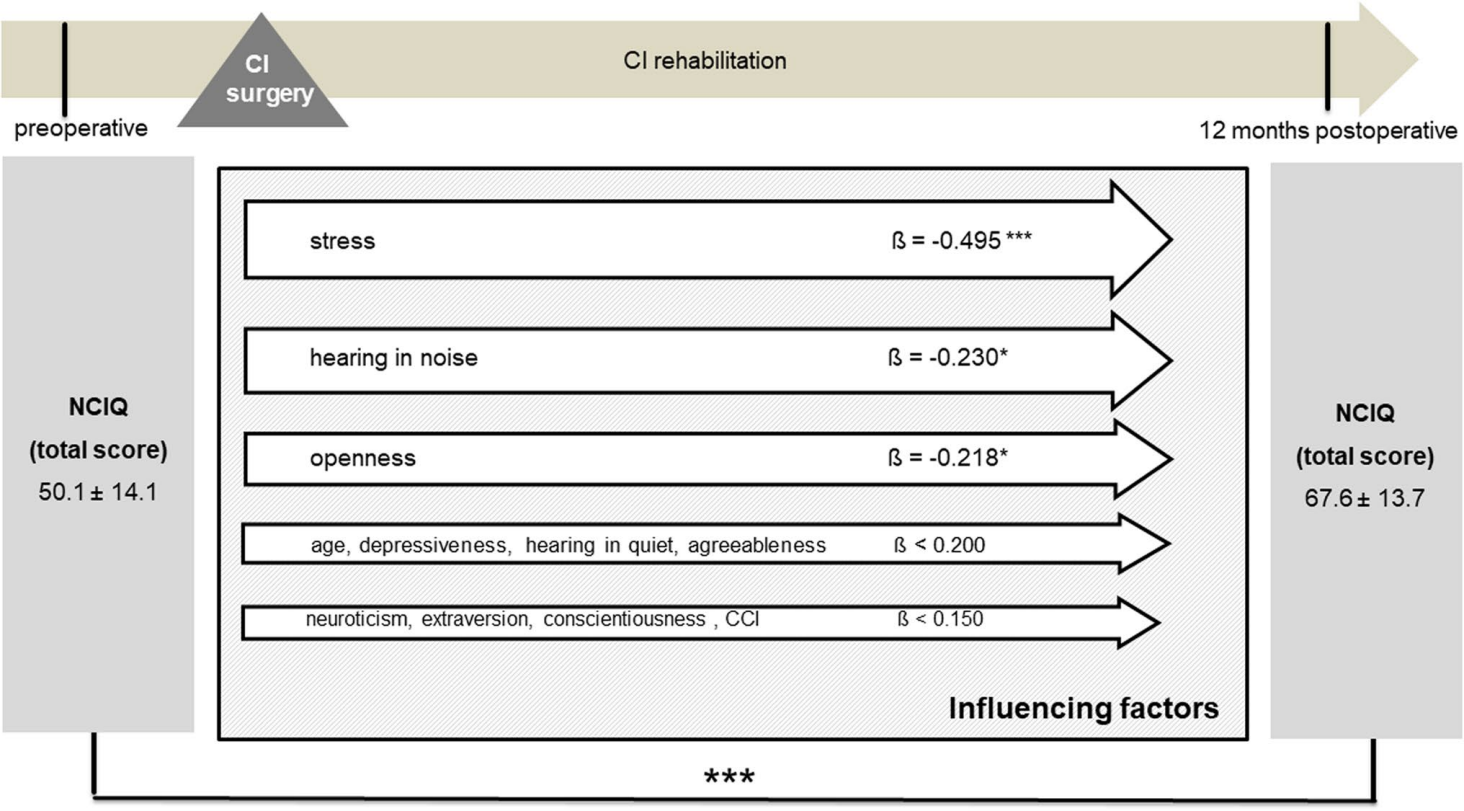
Multiple regression analysis of factors influencing postoperative NCIQ (overall group, *n* = 79); CCI: Charlson Comorbidity Index; Data shown are mean ± SD; ß regression coefficient; ***p ≤ 0.001, *p ≤ 0.05

**Fig. 10:**
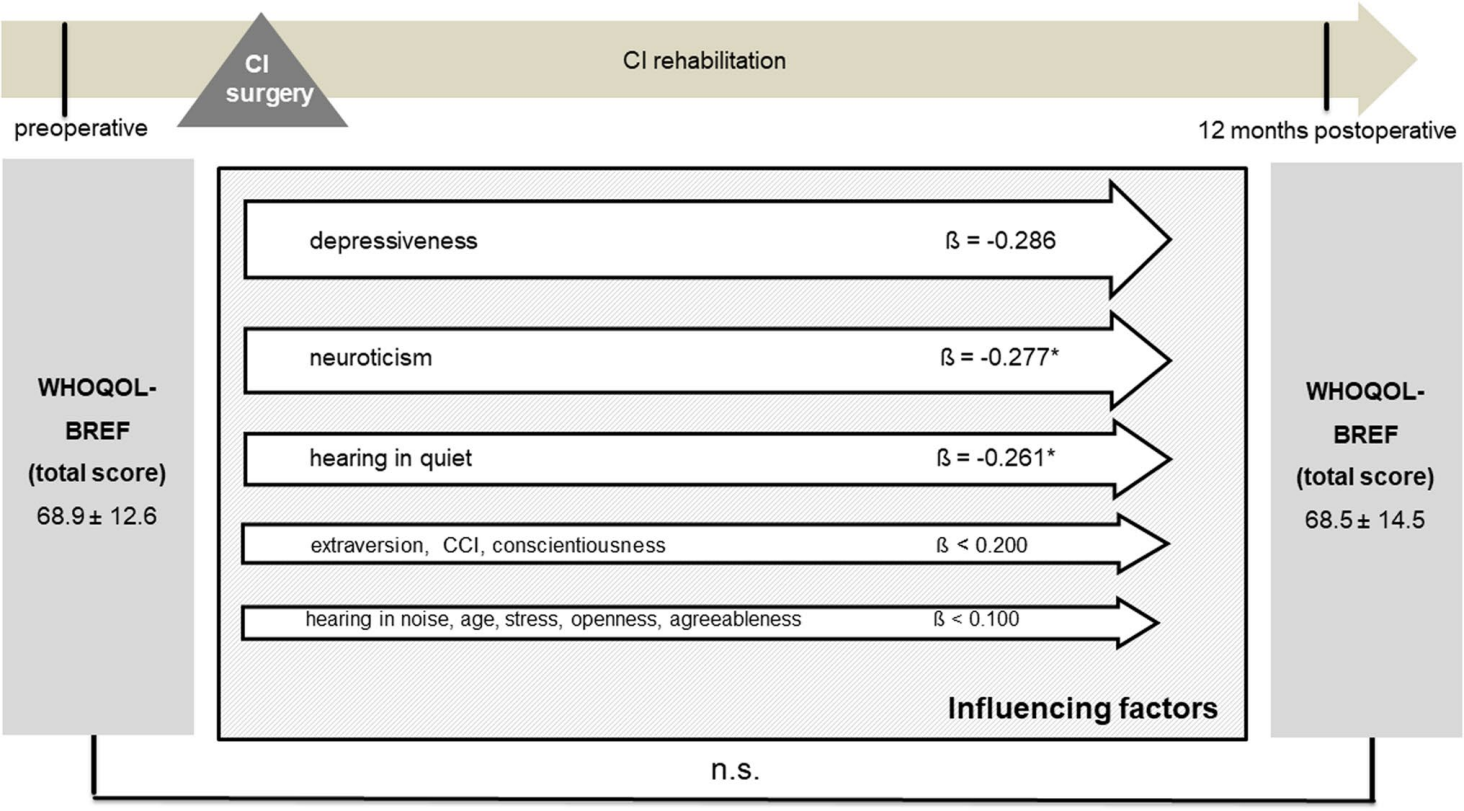
Multiple regression analysis of factors influencing postoperative WHOQOL-BREF (overall group, *n* = 79); CCI: Charlson Comorbidity Index; Data shown are mean ± SD; ß regression coefficient; ***p ≤ 0.001, *p ≤ 0.05, n.s.: not significant (*p* > 0.05)

Multivariate analyses for postoperative disease-specific and generic QOL were performed with explanatory variables, including age, comorbidity, preoperative depressiveness, preoperative stress, preoperative personality dimensions, and postoperative speech perception in quiet and in noise. Due to the stability of personality traits and psychological comorbidities identified, the preoperative scores were included in the analysis here as baseline parameters. The results of the multiple regression analyses showed that, even when adjusting for all mentioned factors preoperative stress, postoperative speech recognition in noise, and the personality trait openness remained significantly associated with NCIQ scores postoperatively (Fig. 9). When evaluating the influence of these factors on the generic QOL, it was found that, when adjusted for the possible influencing factors listed, depressiveness and the personality trait neuroticism had the strongest influence on the generic QOL, along with speech recognition in quiet (Fig. 10).

**Fig. 11:**
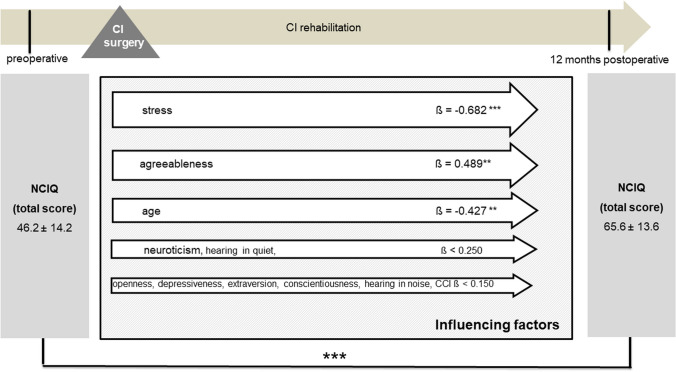
Multiple regression analysis of factors influencing postoperative NCIQ (bilateral hearing loss group, *n* = 45); CCI: Charlson Comorbidity Index; Data shown are mean ± SD; ß regression coefficient; ***p ≤ 0.001, **p ≤ 0.01

**Fig. 12:**
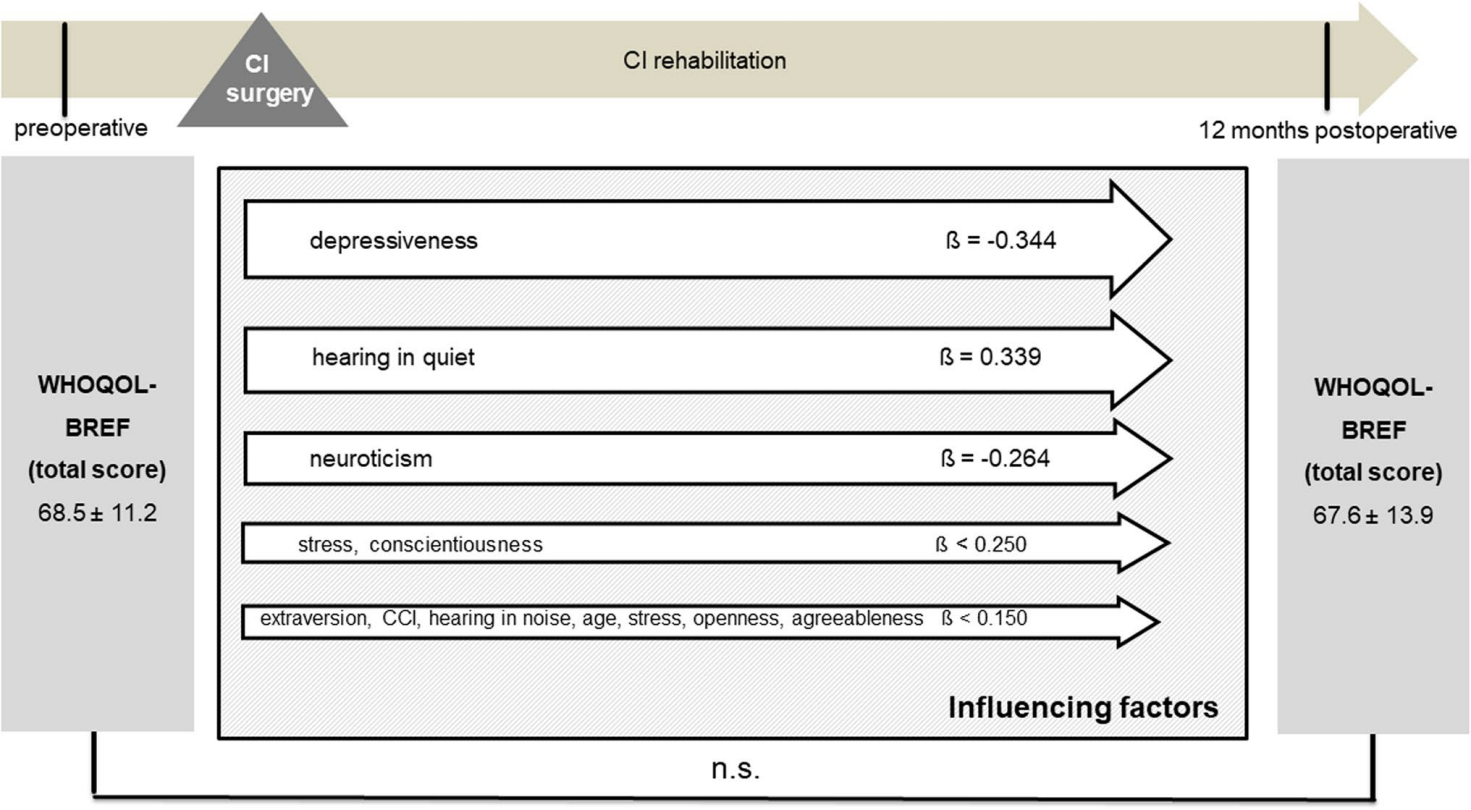
Multiple regression analysis of factors influencing postoperative WHOQOL-BREF (bilateral hearing loss group, *n* = 45); CCI: Charlson Comorbidity Index; Data shown are mean ± SD; ß regression coefficient; n.s.: not significant (*p* > 0.05)

**Fig. 13:**
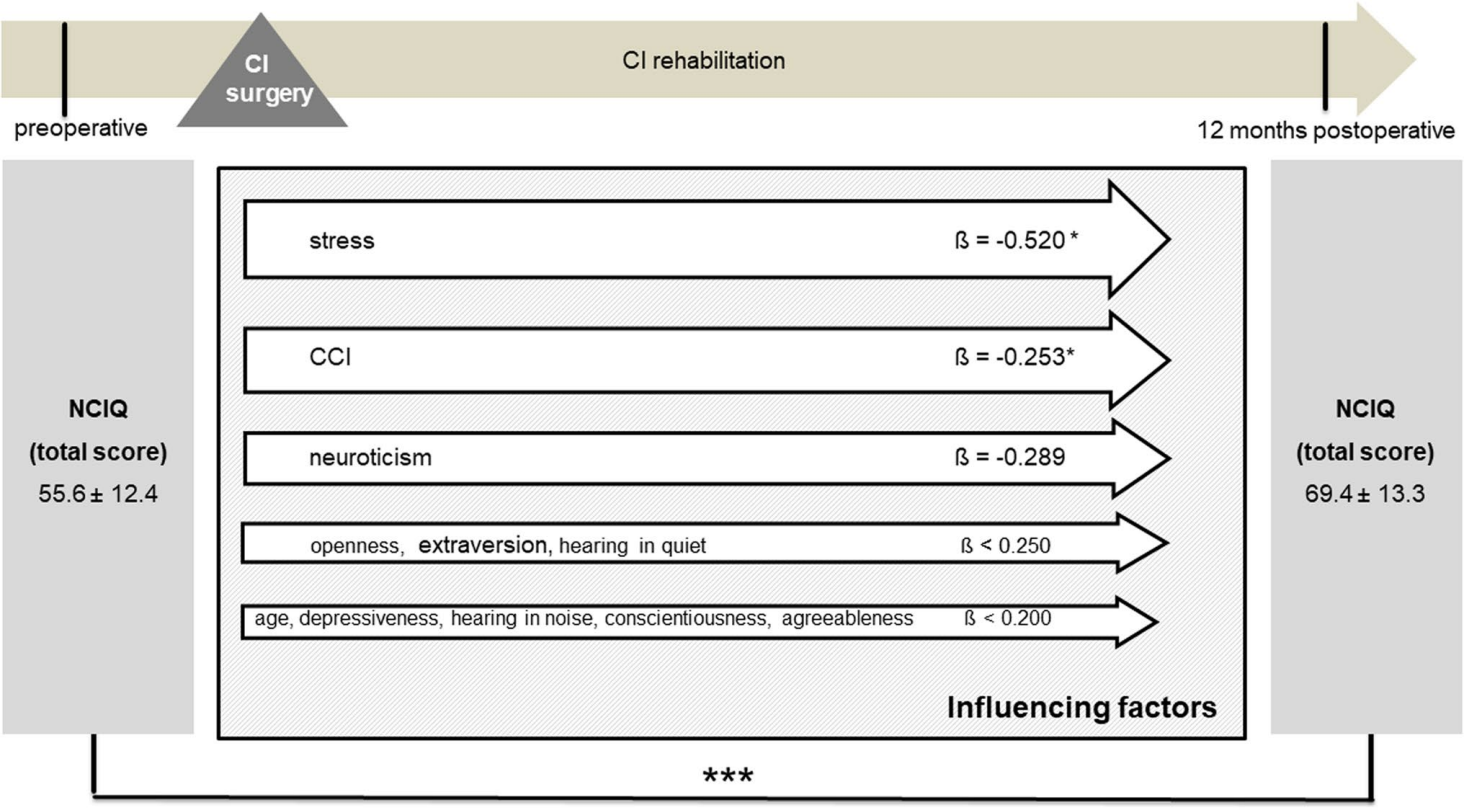
Multiple regression analysis of factors influencing postoperative NCIQ (AHL/SSD group, *n* = 34); CCI: Charlson Comorbidity Index; Data shown are mean ± SD; ß regression coefficient; ***p ≤ 0.001, *p ≤ 0.05

**Fig. 14:**
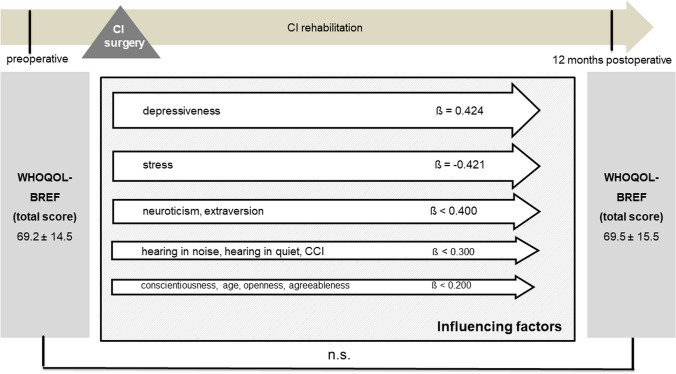
Multiple regression analysis of factors influencing postoperative WHOQOL-BREF (AHL/SSD group, *n* = 34); CCI: Charlson Comorbidity Index; Data shown are mean ± SD; ß regression coefficient; n.s.: not significant (*p* > 0.05)

Multivariate analysis was additionally performed stratified by type of hearing loss for the patient group with bilateral hearing loss (Figs. 11 and 12) and for the AHL/SSD group (Figs. 13 and 14). For both patient groups, the factor stress turned out to be the predominant influencing factor for the disease-specific QOL, according to the overall group, after adjustment for the other factors considered. When considering the generic QOL, depressiveness was the strongest influencing parameter, also stratified by the kind of hearing loss, in accordance with the overall group.

## Discussion

Satisfactory hearing rehabilitation and improvement in quality of life after cochlear implantation have been demonstrated in numerous studies [36, 37]. The positive impact of cochlear implantation on psychological comorbidities or concomitant tinnitus has also been noted in some studies [19, 20, 37, 38]. However, previous research has rarely considered the importance of mental health and personality factors in subjective outcomes. Moreover, in the few studies available, depressiveness and personality have been assessed as individual influencing factors [17, 24]. Therefore, this work aimed to evaluate several potential parameters as influencing factors in a multivariate analysis of a defined patient group.

In Germany, the prevalence of current depressive symptoms is approximately 8.1% (women: 10.2%; men: 6.1%). In our study, the results of the PHQ-9 identified 37 (46.8%) patients with depressive symptoms. Thus, patients with significant hearing impairment appear to have a higher prevalence of depressive disorders and psychological comorbidity, consistent with the findings of previous studies [7, 39–42]. Thomas reported a depression rate of 41% in hearing-impaired patients in 1984n [39]. Knutson & Lansing found a 4.8 times higher rate of depression in patients with profound hearing loss [41]. Sherbourne et al. (2002) even showed a depression rate of 61% in the hearing-impaired group [40]. In a younger group of hearing-impaired patients, a depression rate of 17% was found, with another 30% assigned to the at-risk group [42]. Different prevalence rates can also be attributed to different definitions of depression or divergent cut-off values of the measurement instruments. In our study, a cut-off value of 5 was defined according to the PHQ-D manual. Despite improvement in disease-specific QOL, our study showed no significant improvement in depressiveness. Thus, the results indicate that cochlear implantation cannot be expected to have a generalized positive influence on the mental state of patients. The finding that depressiveness as a multifactorially triggered psychological comorbidity is not affected by the improvement in ear symptoms and hearing function alone was confirmed in comparable studies [13, 43, 44]. The generic quality of life, measured by the WHOQOL-BREF, was stable in the total group during the study period. For our patient group, preoperative and postoperative QOL in the individual subdomains of the WHOQOL-BREF shows worse QOL compared to the general population [45]. Stratified by type of hearing loss, our study showed that the SSD group alone showed improvement in overall QOL. When considering the different hearing loss groups, it was found that the bilaterally hearing-impaired patient group had worse disease-specific QOL preoperatively, which is consistent with further research by Olze et al. (2022) [20]. Postoperatively, no differences were observed after 1 year. Olze et al. (2022) also showed that disease-specific QOL equalized between the groups after 6 months [20].

The hypothesis that depressiveness negatively influences QOL in CI patients could only be partially confirmed within the present analyses. Depressiveness was shown to be the strongest negative influencing factor for overall postoperative QOL measured by WHOQOL-BREF but did not reach the defined significance level, probably due to the multicollinearity between depressiveness and the second strongest influencing factor, neuroticism. Within the analyzed patient group, those with relevant depressive symptoms had significantly worse disease-specific QOL than those without depressive symptoms. However, this effect was diminished after adjustment for somatic comorbidities, personality traits, stress, and audiological performance. Although depressive disorders have been shown to negatively impact QOL in patients with hearing disorders [13, 17], this study showed that for CI patients, the impact appears significantly greater for general QOL than for disease-specific QOL.

Our study identified at least two of five personality traits (neuroticism, openness) as significantly correlated with postoperative QOL. Notably, the personality trait of openness was associated with better postoperative disease-specific QOL. Recent research in this context indicates that patients with hearing impairment have significantly lower scores for openness than patients with normal hearing [24, 46]. Previous studies have shown that personality also plays a crucial role in rehabilitation after medical interventions and that different personality types subjectively rate their QOL differently. A review of 76 English-language studies showed a close correlation between different personality dimensions and the subjective perception of QOL [14]. This review also confirmed that the personality trait openness was associated with better QOL scores, whereas neuroticism was a negative predictor. However, no increase in patients’ openness due to adequate hearing rehabilitation could be shown, consistent with previous studies [24, 47]. When evaluating the association between personality traits and QOL, it should be noted that overlaps exist in the operationalization of the constructs and scale items. Therefore, the association is expected to apply more strongly to the psychosocial than to the physical subscales of the QOL [14]. The present study found that patients with a higher degree of neuroticism rated their general QOL as worse. This could not be demonstrated for disease-specific QOL. Presumably, this is because psychosocial symptoms are more strongly presented in the WHOQOL-BREF than in the NCIQ. It is well known that individuals with high neuroticism are more prone to sadness, anxiety, vulnerability, and negative emotions [48]. Additionally, previous research has highlighted neuroticism as a predictor of depressiveness and perceived stress [25, 26].

Our study found perceived stress to be the strongest negative predictor of disease-specific QOL. Patients with severe hearing impairment show a high degree of suffering regarding stress-related disorders compared to those with normal hearing [49]. Previous research indicated that in hard-of-hearing individuals, the extent of hearing impairment, quality of life, and perceived stress were related to each other and to coping expectations [50]. The mean proportion of CI patients burdened by higher stress perception measured within this study was 31.6%, a slightly increased but nearly identical proportion to a cross-section of the European population during the COVID-19 pandemic (27.41%) [51]. In contrast to previous studies demonstrating the positive effects of cochlear implantation on stress perception, no reduction in stress perception was found in the patient group analyzed here [12, 17, 43, 52]. Since the data collection period also coincided with the peak of the COVID-19 pandemic, it cannot be excluded that the simultaneous requirements for social isolation, mandatory masking, and social restrictions contributed to the constant stress perception and constant depressiveness despite improved hearing. Nevertheless, a survey of 48 adult CI users revealed that the social isolation enforced by the COVID-19 pandemic also had positive aspects for hard-of-hearing people: for example, a calmer environment with less disturbing noise and fewer confrontations with frightening conversational situations [53].

Despite the knowledge of the influence of personality traits and mental health on QOL, minimal research has been devoted to managing psychological distress in patients before/after cochlear implantation. Here, it is important to investigate how (therapeutic) management of psychological comorbidities may influence QOL after surgery. However, our results suggest that modulating stress perception, personality traits, and mood state may provide new methods of improving QOL in patients with CI. The expansion of the therapeutic spectrum and multiprofessionality realized in many centers in recent years is a significant prerequisite for implementing the postulated biopsychosocial model [54]. Psychological counseling and guidance throughout the entire process of CI rehabilitation are thus essential for motivation and a basic attitude toward the therapeutic work as well as hearing impairment generally.

When considering patient age, our study showed that patients in both age groups assessed benefited from cochlear implantation in terms of disease-specific QOL. Previous studies confirmed the success of CI fitting in patient groups aged above 65 years [18, 55, 56]. Analogous to a previous study, multivariate analysis of the overall group showed no significant influence of age on QOL [11]. In another study, better QOL was determined even at older ages compared to the younger patient group [55]. However, a further multivariate analysis considering social aspects showed a negative age effect [10]. In our multivariate analysis, a negative effect on disease-specific QOL was found only for the group of patients with bilateral hearing loss. Thus, based on the data collected as well as previous literature, age cannot be used unreflectively to predict the expected subjective benefit for QOL after implantation. This fact again underlines that no age limits are provided in the current guidelines for cochlear implantation. It should be noted that we used the WHOQOL-BREF for all age groups. The WHOQOL-OLD, which is available especially for the elderly, was not used because it usually has to be handled together with the WHOQOL-BREF, which represents an additional time burden for the elderly patients. The WHOQOL-AGE, which is now available, may be able to circumvent this problem. However, there is currently no validated version available in German and a cross-national validation is pending [57, 58].

Clinical observations show that comorbidities can represent an enormous additional burden for patients. The fact that the additional burden caused by comorbidities sometimes negatively impacts QOL has been described in studies of patients with bronchial and breast carcinoma [27, 28]. Although approximately 43% of the patients in the present study had one or more of the comorbidities assessed with the CCI, no significant influence on disease-specific or general QOL could be determined in the overall patient group. Identical results were also obtained when analyzing the QOL of patients after rehabilitative middle ear surgery [13]. The ambiguous influence of comorbidities on QOL might be due to the heterogeneity of the individual comorbidities assessed with the CCI, the different stages of disease, and the widely varying therapies for the respective concomitant diseases.

Although stress was found to be a significant influencing factor for disease-specific QOL and depressiveness a significant predictor of generic QOL, it should be considered that a bidirectional relationship exists between the factors, and further studies should explore this more deeply. Due to the highly complex and differing definitions of “stress,” many measurement instruments exist for determining stress levels. With the PHQ-D stress module, only one screening instrument, which is also widely used in the clinical setting, was used in the present study. Previous studies have shown that, despite its brevity, this is a reliable measurement instrument that can primarily be processed quickly by patients [59]. The severity of depressiveness was also assessed with a screening instrument, with the PHQ-9 having high sensitivity and specificity for detecting depressive symptoms [33]. Furthermore, we did not consider whether our patients were already undergoing psychotherapeutic or medication-based treatment.

It must be acknowledged that all psychometric recordings are self-reported by patients. No diagnostic interviews or even psychological/psychotherapeutic assessments were performed. The data collection was based on a very heterogeneous group of patients. The age structure, hearing history, and hearing configuration differed and could only be partially incorporated into the influencing factors. We did not include factors in our analysis that could be potential predictors of QOL. For example, we did not specifically analyze the factors of tinnitus, anxiety disorders, social factors, coping, or vestibular symptoms. Based on the psychological influence parameters identified in this study, the model should be expanded to include these factors in a structured manner in follow-up studies.

The present study confirmed that the postoperative satisfaction of patients after cochlear implantation is significantly moderated by psychometric factors. Thus, the importance and potential of multiprofessional counseling during pre-diagnosis and within rehabilitation should be emphasized at this point [3, 4, 60, 61]. Although the current guideline for the therapy and rehabilitation process with CI mentions integrating psychosocial counseling services, these are not yet standard, established components of all therapy and rehabilitation concepts. The results of this study show that an interdisciplinary therapy concept with audiological, medical, hearing therapy, and particularly, psychological components is needed to realize patients’ and practitioners’ expectations regarding CI care and identify possible negative predictors such as psychological disorders. Against this background, the need to integrate appropriate screening instruments into the pre-diagnostic and rehabilitation processes is noted. If psychiatric disorders or distinct personality structures associated with negative traits are detectable with psychometric evaluation questionnaires preoperatively or during postoperative follow-up, interdisciplinary cooperation with outpatient psychiatric or psychosomatic co-care should be encouraged and initiated in future practice.

## Conclusion

Depressive disorders are more prevalent in patients with severe to profound hearing loss. A high expression of the personality trait neuroticism and an additional depressive disorder lead to worse generic postoperative QOL after cochlear implantation. Conversely, disease-specific QOL is mainly influenced by individual stress perception. Therefore, as well as improvements in screening, diagnosis, and treatment of patients with comorbid depressive disorders, assessment of stress levels and personality traits is needed throughout cochlear implantation and rehabilitation. If no improvement in QOL is determined after cochlear implantation despite simultaneous improvement in hearing status, a previously unrecognized depressive disorder, increased stress load, or high or low expression of personality traits should be considered.

## Data Availability

The original data can be provided upon request.
